# Metformin and Thymoquinone Synergistically Inhibit Proliferation of Imatinib-Resistant Human Leukemic Cells

**DOI:** 10.3389/fphar.2022.867133

**Published:** 2022-04-13

**Authors:** Una Glamoclija, Lejla Mahmutovic, Esma Bilajac, Violeta Soljic, Katarina Vukojevic, Mirza Suljagic

**Affiliations:** ^1^ Department of Biochemistry and Clinical Analysis, University of Sarajevo-Faculty of Pharmacy, Sarajevo, Bosnia and Herzegovina; ^2^ Department of Histology and Embryology, School of Medicine, University of Mostar, Mostar, Bosnia and Herzegovina; ^3^ Scientific Research Unit, Bosnalijek JSC, Sarajevo, Bosnia and Herzegovina; ^4^ Genetics and Bioengineering Department, Faculty of Engineering and Natural Sciences, International University of Sarajevo, Sarajevo, Bosnia and Herzegovina; ^5^ Faculty of Health Studies, University of Mostar, Mostar, Bosnia and Herzegovina; ^6^ Department of Anatomy, Histology and Embryology, University of Split School of Medicine, Split, Croatia; ^7^ 3D BioLabs, FabLab Bosnia and Herzegovina, University of Sarajevo, Sarajevo, Bosnia and Herzegovina

**Keywords:** metformin, thymoquinone, leukemia, therapy resistance, combinatorial therapy

## Abstract

Chemotherapy resistance is one of the major challenges in cancer treatment, including leukemia. A massive array of research is evaluating combinations of drugs directed against different intracellular signaling molecules to overcome cancer resistance, increase therapy effectiveness, and decrease its adverse effects. Combining chemicals with proven safety profiles, such as drugs already used in therapy and active substances isolated from natural sources, could potentially have superior effects compared to monotherapies. In this study, we evaluated the effects of metformin and thymoquinone (TQ) as monotherapy and combinatorial treatments in chronic myeloid leukemia (CML) cell lines sensitive and resistant to imatinib therapy. The effects were also evaluated in primary monocytic acute myeloid leukemia (AML) and chronic lymphocytic leukemia (CLL) cells. Both compounds induced a dose- and time-dependent decrease of viability and proliferation in tested cells. Metformin had similar IC_50_ values in imatinib-sensitive and imatinib-resistant cell lines. IC_50_ values of TQ were significantly higher in imatinib-resistant cells, but with a limited resistance index (2.4). Synergistic effects of combinatorial treatments were observed in all tested cell lines, as well as in primary cells. The strongest synergistic effects were observed in the inhibition of imatinib-resistant cell line proliferation. Metformin and TQ inhibited the nuclear factor kappa-light-chain-enhancer of activated B cells (NF-κB) signaling and induced apoptosis in tested cell lines and primary cells. The enhanced effects of combinatorial treatments on the induction of apoptosis were more dominant in imatinib-resistant compared to imatinib-sensitive CML cells. Primary cells were more sensitive to combinatorial treatments compared to cell lines. A combination of 1.25 mM metformin and 0.625 µM TQ increased the levels of cleaved poly (ADP-ribose) polymerase (PARP), decreased the levels of proliferation regulatory proteins, and inhibited protein kinase B (Akt) and NF-κB signaling in primary CLL cells. This study demonstrates that combinatorial treatments of imatinib-resistant malignant clones with metformin and TQ by complementary intracellular multi-targeting represents a promising approach in future studies.

## Introduction

Chemotherapy resistance is a well-known phenomenon resulting in cancer cell insensitivity to the available treatments (Minassian et al., 2019). Several factors can cause cancer resistance. Among others, the most common are 1) metabolic changes of tumors under pressure of chemotherapeutics and development of phenotype overcoming the toxic effects of therapy; 2) tumor microenvironment, especially oxygen level and interaction with adjacent cells (Gatenby and Brown, 2018; Al-Akra et al., 2019; Minassian et al., 2019); 3) tumor heterogeneity and clonal evolution together with mutations and immunological system interactions (Kubuschok and Trepel, 2017); 4) numerous adaptive responses and alternative pathways activated under pressure with specific chemotherapeutics (Gatenby and Brown, 2018); and 5) epigenetic effects (Lue et al., 2015). Several tumors develop multi-drug resistance (MDR) characterized by irresponsiveness to distinct drugs (Gatenby and Brown, 2018) that limits the treatment options of leukemia patients. In chronic myeloid leukemia (CML), the therapeutic efficacy of first-line tyrosine kinase inhibitors (TKIs) such as imatinib, dasatinib, and nilotinib is significantly reduced by resistance mechanisms (Cortes and Deininger, 2006). One of the main causes of imatinib resistance is mutation in the breakpoint cluster region- Abelson murine leukemia viral oncogene homolog 1 fusion gene (BCR-ABL1) kinase (Siegfried and Karni, 2018). Some of the pathways that can be targeted to overcome this resistance are Ras-Raf-MAPK (mitogen-activated protein kinase) and PI3K/Akt/mTOR activated by BCR-ABL1. In respect to this, multi-targeted combinatorial therapy should include inhibitors of heat-shock protein 90 (HSP90), MEK 1/2, phosphorylated protein kinase B (p-Akt), and mTOR (Cortes and Deininger, 2006; Jabbour et al., 2011).

Tumor aggressiveness and resistance development often depend on cancer stem cells (CSCs) and tumor-initiating cells (TICs) (Neuzil et al., 2007; Clarke, 2019). They were first identified in leukemia patients and represent a small subpopulation (0.05–1% of total cancer mass) existing in almost all cancers (Neuzil et al., 2007; Clarke, 2019). In CML, CSCs are resistant to TKIs. It is estimated that they cause relapse in almost 50% of all patients who discontinue the therapy (Chereda and Melo, 2016). While current therapies mainly destroy non-dormant cells (Kangwan et al., 2014), targeting CSCs would be an important approach to overcome tumor resistance and metastasis (Skoda et al., 2019). This could be accomplished through mitochondria targeting because CSCs have very high demands on energy and highly depend on mitochondrial activity (Skoda et al., 2019; García-Heredia and Carnero, 2020). With this aim, anti-diabetic drug metformin (inhibitor of mitochondrial respiratory chain I) could be utilized. It has inhibitory effects in CSCs originating from different cancers (Bednar and Simeone, 2012; Bao et al., 2013). Metformin is the most prescribed anti-diabetic drug in the world used for more than 40 years, thus having a proven safety profile. Numerous studies have shown that metformin decreases cancer risk (Saraei et al., 2019; Leng et al., 2021). Metformin exerts anti-cancerous effects by two distinct mechanisms, including an indirect, insulin-dependent mechanism (Dowling et al., 2011), where metformin sensitizes the cells to insulin, leading to a decrease in glucose and insulin levels in circulation, indirectly diminishing pro-survival activity through insulin receptors. This inhibits commonly activated downstream pathways in cancers, including PI3K/Akt/mTOR and MAPK pathways (Taniguchi et al., 2006). However, many previous studies proposed controversial results regarding metformin effects on p-Akt levels in different cell lines— indicating no effects (Vakana et al., 2011; Shi et al., 2012; Scotland et al., 2013), inhibition (Grimaldi et al., 2012; Rosilio et al., 2013), or even stimulation of Akt (Janjetovic et al., 2011; Grimaldi et al., 2012; Leclerc et al., 2013; Scotland et al., 2013). The second mechanism is insulin independent, through the inhibition of the mitochondrial respiratory chain complex I (Fontaine, 2018) that leads to the activation of AMP-activated protein kinase (AMPK) and inhibition of the mTOR pathway (Rosilio et al., 2014). Metformin interferes with cancer cells’ metabolism and mimics glucose deprivation causing an accumulation of inadequately folded proteins and endoplasmic reticulum stress (Leclerc et al., 2013). This leads to decreased survival of leukemia cells (Rosilio et al., 2013). Despite the various pathways targeted by metformin, certain cancer cells develop resistance to this drug and combinatorial therapy could be a key approach to overcome this resistance (Scotland et al., 2013).

Combinatorial therapy utilizes compounds targeting different signaling pathways with the aim of overcoming cancer resistance and increasing the effectiveness of therapy while decreasing its adverse effects. This approach is already used in CML treatment (Bhaskar et al., 2014; Mu et al., 2021). Combinatorial therapy with metformin and dasatinib in chronic lymphocytic leukemia (CLL) is one of the promising approaches (Marignac et al., 2013). Chemicals with proven safety profiles, such as drugs already used in therapy and active substances isolated from natural sources, could find their applications in combinatorial cancer therapy (Rosilio et al., 2014). Drug repurposing is crucial in the development of combinatorial therapies. With well-known safety profiles supported by data from completed clinical studies and data from clinical practice, approved drugs offer an opportunity for fast development of new combinations. Combining metformin with specific Akt (Scotland et al., 2013; Jang et al., 2021) and nuclear factor kappa-light-chain-enhancer of activated B cells (NF-κB) inhibitors (Mauro et al., 2011) could help decrease the drug concentrations used in therapy and overcome resistance.

Phytochemicals with a known safety profile can be used in combinatorial treatments. For instance, thymoquinone has an inhibitory activity against Akt (Koka et al., 2008; Yi et al., 2008; Arafa et al., 2011; Hussain et al., 2011; Rajput et al., 2013; Khan et al., 2019) and NF-κB (Sethi et al., 2008), representing a good candidate for combinatorial treatment with metformin. TQ transcriptionally up-regulates PTEN in doxorubicin-resistant MCF-7/DOX breast cancer cells leading to a decrease of phosphorylated Akt (Arafa et al., 2011). TQ suppresses NF-κB activation in a time- and dose- dependent manner resulting in a down-regulation of NF-κB–regulated gene products (Zhang et al., 2016). TQ inhibits NF-κB through direct interaction with the p65 subunit and suppression of TNF-induced IKK activation (Sethi et al., 2008). Inhibitory effects of TQ on the fitness of leukemic cells were previously shown in various *in vitro* (El-Mahdy et al., 2005; Alhosin et al., 2010; Dergarabetian et al., 2013; Salim et al., 2013; Khalife et al., 2014; Soltani et al., 2017) and *in vivo* models (Salim et al., 2014; Pang et al., 2017).

However, the mechanism by which metformin and/or TQ interfere with intercellular signaling is not elucidated in the context of leukemic cell resistance.

**FIGURE 1:**
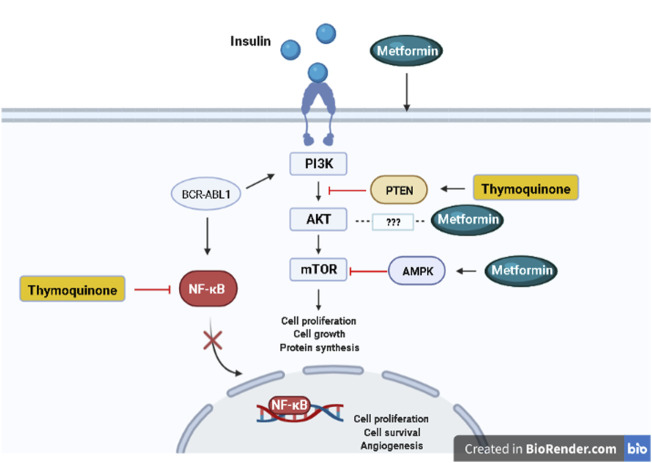
Signaling pathways targeted by metformin and thymoquinone. Created in BioRender.com.

Metformin and TQ combinatorial targeting of complementary pathways in tumor cells represents a novel approach for increasing effectiveness and overcoming resistance to current leukemia therapies (Figure 1).

Considering promising therapeutic potential of metformin and TQ by targeting crucial players in leukemia tumorigenesis, we aimed to evaluate whether metformin and TQ, as monotherapies or in combination will have inhibitory effects on the fitness of primary leukemia cells and CML cell lines sensitive and resistant to imatinib.

## Materials and Methods

### Cell Culture

Cell lines LAMA-84s (CML, a kind gift from Dr. Aleksandar Radujković, University of Heidelberg, Germany), LAMA-84r ((LAMA-84s cell line resistant to 1 µM imatinib (Radujkovic et al., 2005), a kind gift from Dr. Aleksandar Radujković, University of Heidelberg, Germany), and K562 (CML, a kind gift from Dr. Dimitar Efremov, ICGEB, Italy) were cultured in Roswell Park Memorial Institute (RPMI)-1640 basal medium (Sigma-Aldrich, United States), supplemented with 10% Fetal Bovine Serum (FBS), 100 U/ml penicillin, 100 μl/ml streptomycin, 10 mM HEPES, 1 mM sodium pyruvate, and 1% of non-essential amino acid α-Glutamine (Sigma-Aldrich, United States). Additionally, the LAMA-84r growing medium contained 1 µM imatinib. Cell cultures were grown in suspension and maintained at optimal conditions in a humidified atmosphere (95%), 5% CO_2_ at 37°C. Cells were tested for *Mycoplasma* contamination by using the LookOut Mycoplasma qPCR Detection Kit (Sigma-Aldrich, United States).

All experiments with primary cells were performed according to the Declaration of Helsinki. The protocol was approved by the Ethical Committee of the University Clinical Hospital of Mostar (Approval number 426/19). Two peripheral blood samples were obtained from newly diagnosed patients not receiving leukemia therapy. One sample was from a monocytic acute myeloid leukemia (AML) patient and the other from a CLL patient. AML samples were isolated from the bone marrow by density gradient centrifugation on Lymphoprep (1,077 g/ml) (STEMCELL Technologies, Canada). CLL samples were isolated from whole blood by using an EasySep™ Direct Human B-CLL Cell Isolation Kit (STEMCELL Technologies, Canada). The purity of samples was confirmed by flow cytometry, and it was higher than 80 and 90% in AML and CLL samples, respectively. Cells were maintained in a RPMI-1640 complete medium at 37°C and 5% CO_2_ for one day before using them for experiments or freezing.

### Substance Preparation

Metformin (Sigma-Aldrich, United States) was dissolved in 1x phosphate-buffered saline (PBS) (Fisher Bioreagents, United States) as a stock solution of 1 M and filtered through 0.22 µm PTFE filter (Macherey Nagel, Germany) before the usage. TQ (Sigma-Aldrich, United States) was dissolved in DMSO (Sigma-Aldrich, United States) as 100 mM stock solution and was diluted to 1 mM concentration in 1× PBS prior to use. For each experiment, fresh TQ stock solution and appropriate dilutions were prepared. Doxorubicin (Hemofarm, Serbia) was prepared as 100 mM solution in 100% DMSO (Molecular Probes, Life Technologies, United States) and for each experiment, fresh appropriate working concentrations were prepared with the RPMI-1640 complete medium.

### Cell Viability and the Half Maximal Inhibitory Concentration (IC_50_) Determination

LAMA-84s, LAMA-84r, and K562 cells were plated in a 96-well plate in concentration 5 × 10^3^/100 µl of RPMI-1640 complete medium. Cells were treated with a range of metformin (2.5–80 mM) and TQ (2.5–80 µM) concentrations while cells incubated with an appropriate concentration of DMSO or PBS were used as a negative control. After incubation for 48 h, cellular viability was determined by the WST-8 assay using the CCK8 Cell Proliferation Assay Kit (Biotool, United States). 10 µl of WST-8 reagent was added into each well and the absorbance values were measured at 450 nm with the 620 nm reference wavelength on a Multiscan FC microplate reader (Thermo Fisher Scientific, United States). Primary AML and CLL cells were seeded in triplicates in 96-well plates (4 × 10^5^ cells/100 µl), treated with metformin, TQ, and their combinations for 48 h. After the treatment, the WST-8 experiment was performed.

IC_50_ values were calculated by using Calcusyn software (Biosoft, United Kingdom).

### Evaluation of the Effects of Metformin and Thymoquinone on Cell Proliferation

The proliferation of cells was evaluated by the BrdU incorporation assay. LAMA-84s, LAMA-84r, and K562 cells were seeded in triplicates in a 96-well plate with a seeding density of 5 × 10^3^ cells/100 µl and treated with metformin and TQ for 48 h. The quantity of a reaction product was measured by an absorbance at dual wavelengths of 370 and 492 nm using the MultiscanTM GO Microplate Spectrophotometer (Thermo Scientific, United States). The absorbance values were directly proportional to the amount of newly synthesized DNA and a number of proliferating cells.

### Combination Index Calculation

Combination index (CI) calculation by Calcusyn software was used to determine whether two drugs have synergistic, additive or antagonistic effects. The software calculates CI values based on the median-drug effect analysis described by Chou and Talalay (Chou and Talalay, 1984). CI values lower than 0.800 indicate that the compounds have synergistic effects, meaning that the activity of combination has better effects compared to the simple addition of effects of each compound in the combination (Bijnsdorp et al., 2011).

### Apoptosis Evaluation

An assessment of the percentage of cells in early apoptosis, apoptosis, and necrosis was performed by the flow cytometry Annexin V/PI assay. FITC Annexin V Apoptosis Detection Kit I (BD Biosciences, United States) was used according to the manufacturer’s instructions. Briefly, cells were seeded in 6-well plates (seeding density 1 × 10^6^ cells/well). After one hour, the compounds were added into adequate wells. Cells were incubated in a humidified incubator with 5% CO_2_ at 37°C for 48 h prior to the flow cytometry analysis. The cells were washed twice with PBS 1x and then re-suspended in a binding buffer 1x at a concentration 1 × 10^6^ cells/ml. This solution was transferred to a round bottom 5 ml tube. FITC Annexin V and PI were added and incubated 15 min in the dark at room temperature. Binding buffer 1x was added and the flow cytometry analysis on FACSCanto II (BD Biosciences, United States) was performed within 1 hour.

### Western Blot Analysis

Cells were seeded in 6-well plates (seeding density 1 × 10^6^ cells/well) and incubated with compounds for 48 h. Cell lysates were prepared by the RIPA buffer (Sigma-Aldrich, Germany) and the total protein concentrations were determined by the Bradford assay prior to western blot. The signal proteins on the membrane were detected using ECLTM Prime Western Blotting Detection Reagent (GE Healthcare Life Sciences, United Kingdom) representing a horseradish peroxidase substrate for enhanced chemiluminescence (ECL) and signal detection. Protein bands were visualized by Molecular Imager ChemiDocTM XRS + Imaging System (Bio-Rad, United States) and normalized using the Image Lab software (v.6.0). Antibodies were purchased from Cell Signaling Technology, Netherlands. Primary antibodies used were PARP Antibody #9542, Phospho-PKC (pan) (zeta Thr410) (190D10) Rabbit mAb #2060, Phospho-p70 S6 Kinase (Thr421/Ser424) Antibody #9204, Phospho-NF-κB p65 (Ser536) (93H1) Rabbit mAb #3033, Phospho-Akt (Ser473) (D9E) XP^®^ Rabbit mAb #4060, PTEN (D4.3) XP^®^ Rabbit mAb #9188, Mcl-1 Antibody #4572, and Cyclin D1 Antibody #2922. For loading control, β-Actin (8H10D10) Mouse mAb #3700 primary antibody was used. Secondary antibodies anti-rabbit IgG, HRP-linked Antibody #7074, and anti-mouse IgG and HRP-linked Antibody #7076 were used.

### Statistical Analysis

IBM SPSS statistics version 23 (IBM Corporation, United States) was used for statistical analysis. The experiments were repeated at least three times. The Shapiro–Wilk test was used for testing the normality of data. Levene’s test was used for testing homogeneity of variances. In all cases, assumptions were met for one-way ANOVA analysis. Tukey’s post-hoc test was used in cases where Levene’s test indicated homogeneity of variances; otherwise, Games-Howell post-hoc test was used. *p* < 0.05 was considered as the level of statistical significance with the next levels presented through the text: *p < 0.05; **p < 0.01; and ***p < 0.001.

## Results

### Metformin and Thymoquinone Decrease the Viability of Leukemia Cell Lines and Primary Cells

Metformin and TQ monotherapies caused a concentration-dependent decrease of cell viability in treated cell lines and primary cells. Effects of metformin and TQ shown were at mM and µM levels, respectively (Table 1). No statistically significant difference was seen between IC_50_ values of metformin for LAMA-84s and LAMA-84r cell lines. The K562 cell line had significantly higher IC_50_ values compared to LAMA-84 cell lines (*p* < 0.001). LAMA-84r cells (resistant to 1 µM imatinib) were significantly less sensitive to TQ treatment than LAMA-84s (*p* < 0.001). Significantly higher TQ IC_50_ in K562 cell lines compared to LAMA-84 cell lines was observed (*p* < 0.001).

**TABLE 1:** IC_50_ values of metformin and thymoquinone (TQ) after 48 h treatment in leukemia cells, as determined by the WST-8 assay.

Cells	Metformin IC_50_ (mean ± SD) mM	TQ IC_50_ (mean ± SD) µM
LAMA-84s	30.3 ± 1.5	11.8 ± 0.9
LAMA-84r	23.7 ± 5.3	28.6 ± 2.1
K562	61.4 ± 10.2	53.5 ± 3.3
Primary cells, acute monocytic leukemia	13.9	27.1
Primary cells, chronic lymphocytic leukemia	27.7	9.1

### Metformin and Thymoquinone Decrease the Proliferation of Leukemia Cell Lines

**FIGURE 2:**
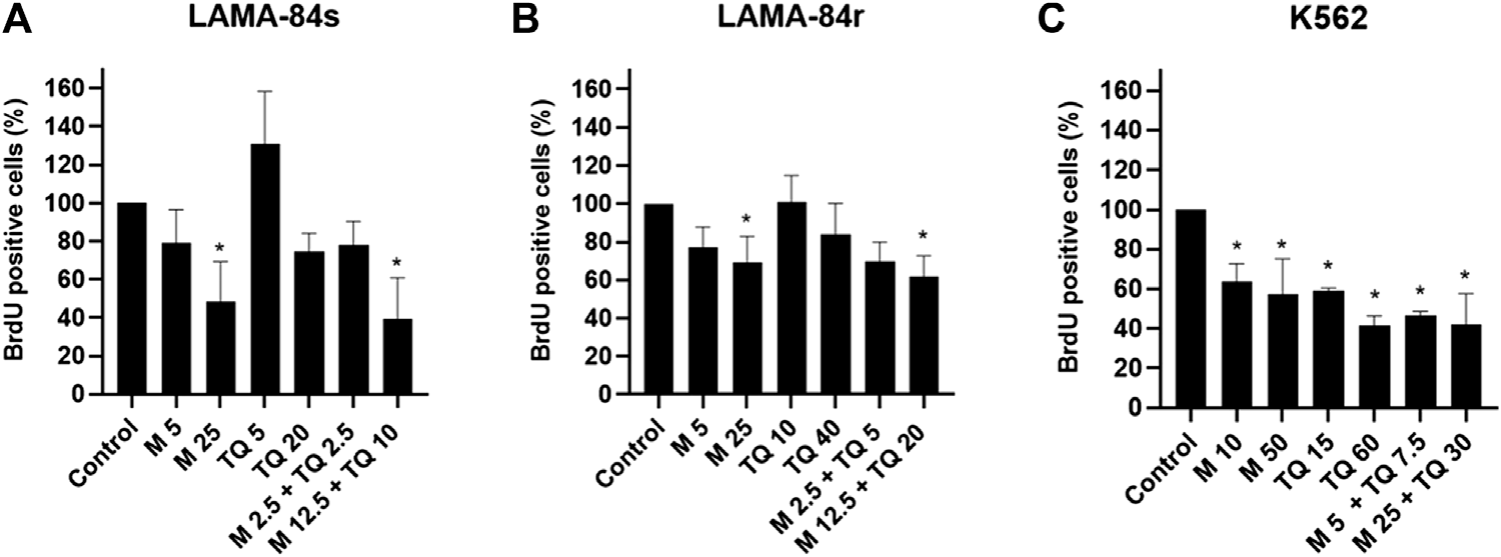
Metformin (M), thymoquinone (TQ), and their combinatorial treatments applied for 48 h caused changes in proliferation as determined by the BrdU assay in **(A)** LAMA-84s, **(B)** LAMA-84r, and **(C)** K562 cell lines. Milimolar (mM) concentrations of metformin and micromolar (µM) concentrations of TQ were applied depending on the cell line as indicated at the X-axis of each graph. Data are presented as mean ± standard deviation. *p < 0.05.

In LAMA-84s and LAMA-84r cells, significant anti-proliferative effects were observed after treatment with metformin monotherapy and higher concentration of applied combination (Figures 2A,B). TQ in a low concentration slightly induced the proliferation of LAMA-84s cells, while a higher TQ concentration decreased cell proliferative rate (Figure 2A). In K562 cell line both metformin and TQ mono and combinatorial treatments incubated for 48 h caused a significant inhibition of cell proliferation (Figure 2C).

### Metformin and Thymoquinone Have Synergistic Effects in Leukemia Cell Lines and Primary Cells

When metformin and TQ were applied in combinatorial treatments, concentrations and protocols of application influenced the obtained CI values (Table 2). Low concentrations of TQ, with stimulatory effects as monotherapy, caused antagonistic effects in combination with metformin. Generally, sequential application of compounds (first TQ for 12 h and then metformin in addition to TQ for 48 h) provided a better synergistic outcome, when compared to the simultaneous application of drugs. The strongest synergistic effect was observed on LAMA-84r cells’ proliferation. When 2 mM metformin and 2 µM TQ were applied simultaneously for 48 h in primary AML and CLL cells, synergistic effects on viability inhibition were observed (Table 2).

**TABLE 2:** Combination index (CI) values on leukemia cell lines as determined by WST-8 and BrdU incorporation assays.

Cell line	Treatment	CI values (WST-8), 48 h treatment	CI values (BrdU), 48 h treatment	CI values (BrdU), 72 h treatment
LAMA-84s	Simultaneous	1.272 ± 0.322	0.622 ± 0.240	0.262 ± 0.024
	Sequential	0.443 ± 0.031	—	—
LAMA-84r	Simultaneous	1,860 ± 1,039	0.314 ± 0.100	0.151 ± 0.075
	Sequential	0.515 ± 0.131	—	—
K562	Simultaneous	0.636 ± 0.038	0.457 ± 0.198	0.384 ± 0.002
	Sequential	0.494 ± 0.066	—	—
AML primary cells	Simultaneous	0.770	—	—
CLL primary cells	Simultaneous	0.630	—	—

AML, acute monocytic leukemia; CLL, chronic lymphocytic leukemia.

### Metformin and Thymoquinone Induce Apoptosis in Leukemia Cell Lines

**FIGURE 3:**
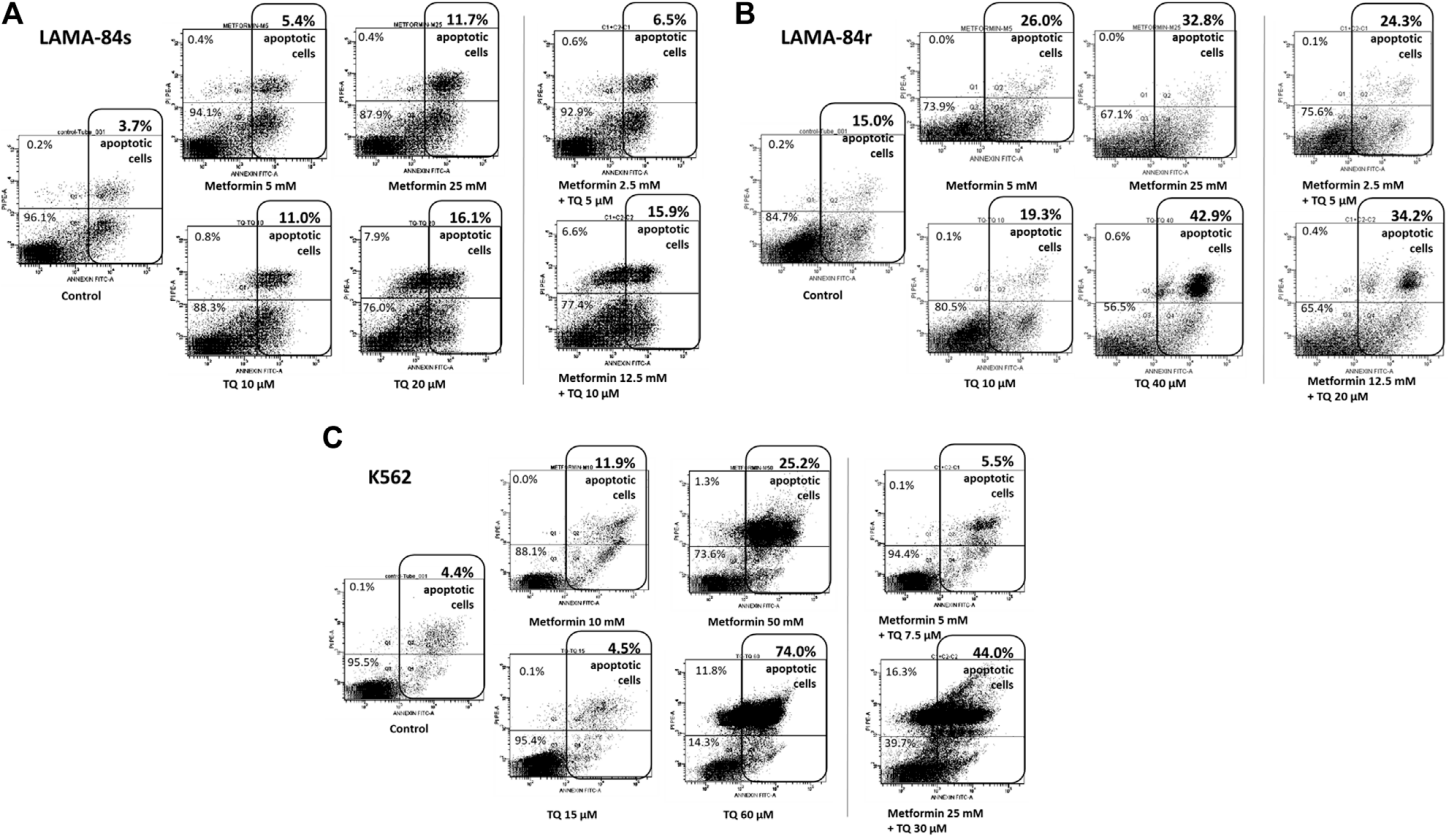
Flow cytometry Annexin V/PI analysis was used to determine the percentage of cells in apoptosis after metformin and thymoquinone (TQ) monotherapies and combinatorial treatments for 48 h in **(A)** LAMA-84s, **(B)** LAMA-84r, and **(C)** K562 cell lines.

n imatinib-sensitive LAMA-84s cells, metformin and TQ have shown mild pro-apoptotic effects (Figure 3A), while induction of apoptosis was observed in imatinib-resistant LAMA-84r cells treated with monotherapies and combinations of metformin and TQ (Figure 3B). LAMA-84s cells treated with a combination of 2.5 mM metformin and 5 µM TQ had 6.5% of apoptotic cells compared to 3.7% apoptotic cells in control. On the other hand, LAMA-84r cells treated with the same combination of metformin and TQ had 24.3% of apoptotic cells compared to 15.0% apoptotic cells in control.

In the K562 cell line, apoptosis was also induced by metformin and TQ monotherapies and combinatorial treatments (Figure 3C).

### Metformin and Thymoquinone Inhibit NF-kB Signaling and Induce Apoptosis in Leukemia Cell Lines and Primary Cells

**FIGURE 4:**
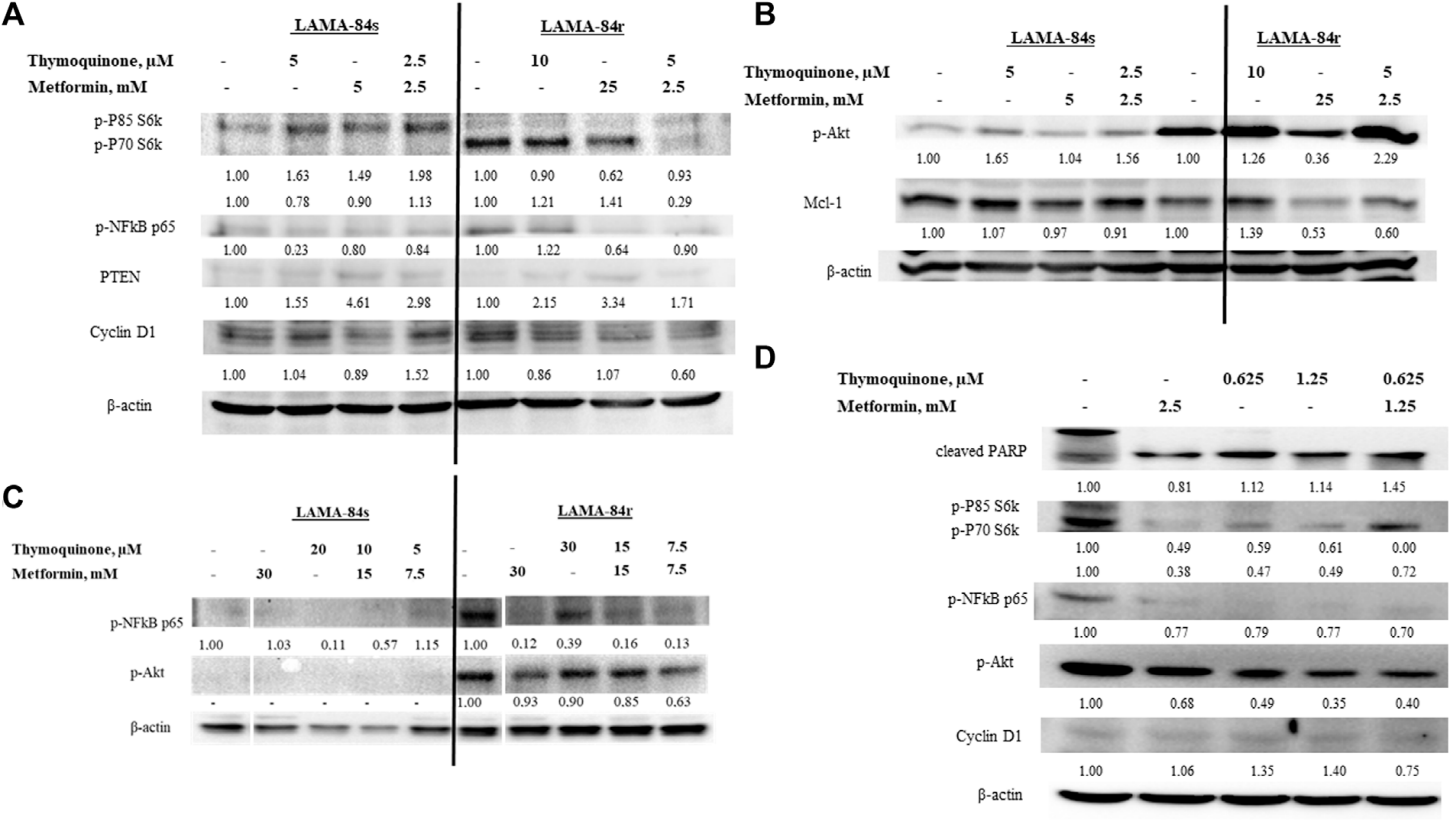
Western blot of cell lysates from **(A)** LAMA-84s and **(B)** LAMA-84r cells treated for 48 h with metformin(M), thymoquinone (TQ), and their combinations, **(C)** LAMA-84s and LAMA-84r cells treated for 48 h with metformin, TQ, and their combinations with 1 µM doxorubicin pretreatment for 48 h, and **(D)** primary CLL cells treated for 48 h with metformin, TQ and their combinations. Numbers represent fold change compared to control. Abbreviations: myeloid cell leukemia-1 (Mcl-1); phosphorylated Akt (p-Akt); Poly (ADP-ribose) polymerase (PARP); phosphorylated NF-κB (p-NF-κB); Phosphatase and tensin homolog (PTEN); phospho-p85 S6 kinase (p-P85 S6k); phospho-p70 S6 kinase (p-P70 S6k); phosphorylated protein kinase C (p-PKC).

LAMA-84r, resistant to 1 µM imatinib, had increased basal levels of p-Akt and p-NF-κB p65, while the basal levels of both kinases were low in LAMA-84s cells. In both cell lines, PTEN levels were low. Metformin treatment decreased the levels of p-Akt and p-NF-κB p65 and slightly increased the level of PTEN in LAMA-84r cells. When low concentration combinatorial treatments were applied, increased p-Akt levels were observed in both LAMA-84r and LAMA-84s cell lines. However, both cell lines had a slightly induced PTEN production (Figures 4A,B). In both cell lines, low concentration combinatorial treatments decreased p-NF-κB p65 levels (Figure 4A).

Results obtained by the BrdU assay showing that low combinatorial concentrations of metformin and TQ had synergistic effects in the inhibition of LAMA-84r cells proliferation were confirmed by western blot. Levels of proteins involved in proliferation, including cyclin D1, phospho-p85 S6 kinase (p-P85 S6k), and phospho-p70 S6 kinase (p-P70 S6k), were decreased when the low concentration combinatorial treatment was applied. However, the opposite effects were seen in LAMA-84s cells (Figure 4A).

Induction of apoptosis in LAMA-84r and mild pro-apoptotic effects in LAMA-84s cells by the combinatorial treatment with metformin and TQ observed in flow cytometry experiments were confirmed by Mcl-1 levels detected in western blot (Figure 4B).

With the aim of Akt phosphorylation induction, LAMA-84s and LAMA-84r cells were pre-treated with 1 µM doxorubicin for 48 h and after that, metformin and TQ were added. However, in LAMA-84s cells, p-Akt and p-NF-κB levels remained low. In LAMA-84r cells pre-treated with doxorubicin, mono and combinatorial treatments with metformin and TQ caused an inhibition of Akt and NF-κB phosphorylation (Figure 4C).

Primary CLL cells were more sensitive, compared to cell lines, to the effects of metformin and TQ in both, mono and combinatorial treatments. A very low combinatorial concentration (1.25 mM metformin + 0.625 µM TQ) increased the levels of cleaved poly (ADP-ribose) polymerase (PARP), decreased levels of proteins involved in proliferation, and caused the inhibition of Akt and NF-κB phosphorylation (Figure 4D).

## Discussion

In this study, mono and combinatorial treatments with metformin and TQ caused a concentration-dependent decrease of viability and proliferation and induction of apoptosis in leukemia cells. Stronger synergistic effects on proliferation of imatinib-resistant compared to imatinib-sensitive CML cells were observed. Also, stronger effects of combination on the induction of apoptosis were seen in resistant compared to sensitive CML cells. Primary cells were more sensitive to combinatorial treatments compared to cell lines.

Metformin and TQ monotherapies had dose-dependent effects on leukemia cells. Similar to previous studies, metformin had millimolar (mM) IC_50_ values (Table 3) while TQ had micromolar (µM) IC_50_ values (Table 4). Results obtained in cell lines were confirmed in primary cells from one AML and one CLL patient. AML and CLL represent two of the most common leukemia types in adults (Salim et al., 2014) and it was of particular interest to evaluate the effects of metformin, TQ, and their combination in primary patient samples. The IC_50_ value for metformin in primary CLL cells and leukemia cell lines was between 13.9 and 30.3 mM (Table 1). The exception was observed with K562 cell lines which was less sensitive to metformin. Voltan et al., 2016 have found that metformin treatment for 48 h had similar effects in leukemic cell lines (EHEB and JVM-2) and primary CLL cells with IC_50_ values 11.6 and 10.2 mM, respectively (Voltan et al., 2016). Primary AML cells were more sensitive to metformin treatment with IC_50_ 13.9 mM. Energy metabolism plays an important role in the therapy response of AML cells (Scotland et al., 2013). AMPK is functional in AML and can be activated by metformin, leading to mTOR inhibition and a significant decrease of proliferation (Green et al., 2010). The inhibitory activity of metformin in leukemia primary cells was confirmed by different authors. Shi et al., 2015 have found that primary Ph + ALL blasts are more sensitive to metformin treatment than K562 cell lines (Shi et al., 2015). Vakana et al. have found that metformin caused the inhibition of colony formation of primary CML cells. The effects were already seen at 1 mM concentration while 10 mM concentration had similar effects to imatinib (Vakana et al., 2011). TQ IC_50_ in primary AML cells (27.1 µM) is in accordance with results previously obtained on U937 AML cell line (21.4 µM) (Khalife et al., 2014). This is the first study to show TQ effects in CLL primary cells, where we have observed a complete loss of viability at 10 µM concentration.

**TABLE 3:** Half maximal inhibitory concentration (IC_50_) values from the literature for metformin in leukemia cell lines.

Cell line	Cell line description	Incubation period	Method used	Metformin IC_50_ (mM)	Reference
K562	CML	5 days	MTT assay	5.0	Vakana et al. (2011)
K562	CML	72 h	MTT assay	7.5	Shi et al. (2015)
K562R	CML resistant to 1 µM imatinib	72 h	MTT assay	1.1	Shi et al. (2015)
SUP-B15	Ph + ALL	72 h	MTT assay	26.1	Shi et al. (2015)
Primary Ph + ALL	Primary Ph + ALL blasts	72 h	MTT assay	6.9	Shi et al. (2015)
Jurkat	T-ALL	48 h	MTT assay	5.6	Grimaldi et al. (2012)
Jurkat	T-ALL	72 h	MTT assay	20.8	Shi et al. (2012)
HL-60	AML	24 h/48 h/72 h	CCK8 (WST-8) assay	33.1/15.2/10.4	Yuan et al. (2020)
THP-1	AML	24 h/48 h/72 h	CCK8 (WST-8) assay	78.8/12.0/6.4	Yuan et al. (2020)
KG-1	AML	48 h	CCK8 (WST-8) assay	11.9	Zhou et al. (2021)
Kasumi-1	AML	48 h	CCK8 (WST-8) assay	10.5	Zhou et al. (2021)
THP-1	AML	48 h	CCK8 (WST-8) assay	11.2	Zhou et al. (2021)
KG-1	AML	24 h/72 h	XTT assay	40.0/7.7	Valiulienė et al. (2021)
KG-1A	AML resistant to	24 h/72 h	XTT assay	46.3/6.6	Valiulienė et al. (2021)
NB-4	AML	24 h/72 h	XTT assay	43.0/3.6	Valiulienė et al. (2021)

ALL, acute lymphoblastic leukemia; CML, chronic myeloid leukemia; Ph +, Philadelphia chromosome positive.

**TABLE 4:** Half maximal inhibitory concentration (IC_50_) values found in the literature for thymoquinone (TQ) in leukemia cell lines.

Cell line	Cell line description	Incubation period	Method used	TQ IC_50_ (µM)	Reference
Jurkat	T-ALL	24 h	MTS assay	24.3	Alhosin et al. (2010)
Jurkat	T-ALL	24 h	MTS assay	19.5	Soltani et al. (2017)
Jurkat	T-ALL	48 h	MTS assay	17.3	Soltani et al. (2017)
Jurkat	T-ALL	72 h	MTS assay	14.1	Soltani et al. (2017)
Jurkat	T-ALL	48 h	MTT assay	28.0	Dergarabetian et al. (2013)
U937	AML	24 h	WST-1 assay	31.3	Khalife et al. (2014)
U937	AML	48 h	WST-1 assay	21.4	Khalife et al. (2014)
CEM	T-ALL	48 h	MTT assay	8.0	Dergarabetian et al. (2013)
CEMss	T-ALL	24 h	MTT assay	6.1	Salim et al. (2013)
MT-2	T-ALL HTLV-1 positive	48 h	MTT assay	35.0	Dergarabetian et al. (2013)
HuT-102	T-ALL HTLV-1 positive	48 h	MTT assay	85.0	Dergarabetian et al. (2013)
HL-60	Myeloblastic leukemia	24 h	MTT assay	23.0	El-Mahdy et al. (2005)

AML, acute monocytic leukemia; ALL, acute lymphoblastic leukemia; HTLV-1, human T-lymphotropic virus 1.

Similar effects of metformin in 1 µM imatinib-sensitive and imatinib-resistant CML cell lines (LAMA-84s and LAMA-84r) indicate that metformin could be used in overcoming resistance to current CML therapies. Vakana et al., 2011 were first to show that metformin have an inhibitory activity against Ph + cell lines and primary cells, including those carrying T315I-BCR-ABL mutation rendering them resistant to imatinib therapy (Vakana et al., 2011). Shi et al., 2015 have found that K562 cells resistant to 1 µM imatinib were more sensitive to metformin treatment when compared to imatinib-sensitive K562 cells. Metformin had synergistic effects with imatinib in Ph + cells (Ph + ALL SUP-B15 cell line, Ph + ALL primary blasts, K562 cells resistant, and K562 sensitive to 1 µM imatinib) (Shi et al., 2015). Metformin had similar effects in KU812 CML cells sensitive and resistant to imatinib therapy (Reddy et al., 2013) while in K562-Lucena multi-drug resistant cells, metformin decreased the levels of P-glycoprotein (P-gp) and had synergistic effects with imatinib (Curvello et al., 2013). P-gp is a surface glycoprotein which plays an important role in the development of multi-drug resistance (Dewanjee et al., 2017). LAMA-84r cell line used in this study is characterized by an over-expression of BCR-ABL and increased the activity of P-gp (Radujkovic et al., 2014), so metformin could decrease P-gp levels and have a cytotoxic activity in this cell line. Effects of metformin against resistant Ph + cells need to be further investigated with a focus on leukemia initiating cells. The obtained data so far, including results of this study, are supportive for the potential application of metformin in abolishing leukemia resistance and inhibiting leukemia initiating cells. LAMA-84r cells were less sensitive to TQ than LAMA-84s cells with IC_50_ values 28.6 ± 2.1 μM and 11.8 ± 0.9 μM, respectively. However, the TQ resistance index in LAMA-84r cells is only 2.4. Resistance index is a value obtained by the division of IC_50_ of resistant cells and IC_50_ of same cells sensitive to certain agents (Scappini et al., 2004). Usually, cells designated to be resistant to certain therapies have much higher resistance index such as 744.0 for bosutinib, 692.0 for nilotinib, or 360.0 for dasatinib in CML cells harboring T315I mutation (Sang et al., 2016), 9.1 for imatinib in resistant K562 cells (Sang et al., 2016), 13.3 and >20 for imatinib in 1 µM resistant KBM7 and KBM5 CML cell lines, respectively (Scappini et al., 2004), and 9.6 for doxorubicin in certain lymphoma subpopulations (Zhang et al., 2018). Previously, it has been shown that TQ has a potential to overcome resistance in different cancer cells, especially when combined with other therapeutics (Arafa et al., 2011; Siveen et al., 2014). Our results demonstrated that TQ and metformin have synergistic effects in the inhibition of metabolic activity and proliferation of LAMA-84r cells.

K562 was significantly less sensitive to treatment with metformin and TQ. Although LAMA-84 and K562 cell lines are both Ph + CML cells, their phenotypes are different. LAMA-84 has elevated proteins associated with an invasive behavior, while K562 has elevated proteins connected to the development of resistance. Some of the expressed proteins found in K562 but not in LAMA-84 cells are Annexin A1 involved in exocytosis, glutathione S-transferases involved in detoxification, and heat-shock proteins (HSP27 and HSP70) with cytoprotective effects (Fontana et al., 2007). Those proteins could contribute to a more resistant phenotype against metformin and TQ treatment. HSP70 is recognized as a protein involved in imatinib resistance, and indeed, in preliminary experiments we have found that in LAMA-84s cells viability decreased dramatically after2 days in 1 µM imatinib, while K562 cells were less sensitive and about 20% could survive 40 days in 1 µM imatinib (data not shown).

Strong inhibitory effects of metformin on AML cell lines’ proliferation have previously been shown (Scotland et al., 2013; Gopalakrishnapillai et al., 2014). Grønningsæter et al. found that metformin inhibited proliferation in each of the 17 patients’ primary AML cells *in vitro* (Grønningsæter et al., 2020). In our experiments, metformin caused a significant inhibition of proliferation on both imatinib-sensitive and imatinib-resistant cell lines, as shown by the BrdU assay (Figure 2). On the other side, TQ effects were depending on dose and time of incubation. A mild induction of LAMA-84s cells’ proliferation by a low dose of TQ was probably due to compensatory mechanisms that were overcome when higher concentrations of TQ were applied. Opposite effects of lower and higher concentrations (hormetic effects) are known for various compounds (Calabrese and Mattson, 2017). Thymol, a compound that is transformed to TQ by catalytic oxidation of essential oils (Jukić and Miloš, 2005), was found to be protective or cytotoxic depending on the cell line used and applied concentration (Günes-Bayir et al., 2020). Previous studies have demonstrated anti-proliferative effects of TQ in the T-ALL Jurkat cell line, starting from a 10 µM concentration (Alhosin et al., 2010) and malignant T-lymphocytes (Diab-Assaf et al., 2018). Combinatorial treatment with metformin and TQ significantly inhibited proliferation of LAMA-84r cells as shown by the BrdU assay (Figure 2B) and decreased levels of cyclin D1, phospho-p85 S6 kinase (p-P85 S6k), and phospho-p70 S6 kinase (p-P70 S6k) (Figure 4A). In LAMA-84s cells, lower combinatorial concentrations increased levels of proliferation proteins (Figure 4A), while a higher combinatorial concentration inhibited proliferation (Figure 2A). The observed hormetic effects of TQ applied in CML cell lines as monotherapy or in combination with metformin need to be further evaluated. Those effects were not observed in primary cells.

CI values obtained in combinatorial studies depended on the cell line, concentrations of used compounds, as well as the procedure for their application. Synergistic effects were observed in all cell lines regarding a decrease of viability and inhibition of proliferation. The strongest synergistic effects were observed when cells were pre-treated with TQ for 12 h, and then co-treated with metformin for an additional 48 h (Table 2). Previous studies have found the strongest synergism when TQ was applied before topotecan (Khalife et al., 2014) and gemcitabine (Mu et al., 2015). In the U937 AML cell line, TQ in combination with topotecan showed synergistic anti-proliferative and pro-apoptotic effects with pre-exposure to TQ more effective than simultaneous application (Khalife et al., 2014). Also, in the same AML cell line, pretreatment with Akt inhibitor resulted in strong synergistic effects with metformin (Scotland et al., 2013). What we found particularly interesting is the fact that sequential treatment had strong synergistic effects (CI value 0.515 ± 0.131) in LAMA-84r cells, resistant to 1 µM imatinib. The strongest synergistic effects were observed in LAMA-84r cell line proliferation where CI values for 48 and 72 h’ treatments were 0.314 ± 0.100 and 0.151 ± 0.075, respectively. Less intensive synergistic effects were also observed in proliferation decrease of imatinib-sensitive cell lines (Table 2). It is important to note that LAMA-84r cells were grown in a medium containing imatinib and stronger synergistic effects could potentially be due to this additional compound. This result needs to be further explored.

Metformin and TQ mono and combinatorial therapies induced an apoptosis in tested cell lines. It was previously shown that TQ had pro-apoptotic effects in CML (Sethi et al., 2008), ALL (Alhosin et al., 2010; Dergarabetian et al., 2013; Salim et al., 2013; Soltani et al., 2017), myeloblastic leukemia (El-Mahdy et al., 2005), and AML (Khalife et al., 2014) cells. The metformin pro-apoptotic effect depends on the dominant energy production pathway and compensatory capacity of treated cells (Scotland et al., 2013). In LAMA-84s cells, a slight increase in the percentage of apoptotic cells was observed with combinatorial therapies, and this was confirmed by decreased levels of Mcl-1 protein in western blot experiments. In LAMA-84r cells, similar effects were seen, but were more pronounced when compared to LAMA-84s cells. The relatively high level of apoptosis (15.0%) in controls of LAMA-84r cells was probably due to 1 µM imatinib present in the growth medium for this cell line. Stronger effects of combination on induction of apoptosis were seen in resistant (2.5 mM metformin + 5 µM TQ vs. control, 15.0% vs. 24.3%) compared to sensitive CML cells (2.5 mM metformin + 5 µM TQ vs. control, 3.7% vs. 6.5%). In LAMA cells resistant to imatinib therapy, 25 mM metformin monotherapy or a ten times decreased concentration (2.5 mM) of metformin in combination with 5 µM TQ reduced levels of Mcl-1 to 0.53 and 0.60 compared to control, respectively (Figure 4B). Inhibition of Mcl-1 by metformin was previously shown in AML cells (Zhou et al., 2021) and other tumors (Park et al., 2018; Ye et al., 2020; Chen et al., 2021).

Our results show that metformin and TQ treatments for 48 h decrease levels of p-NF-κB p65 in LAMA-84 cells sensitive and resistant to imatinib, as well as in primary CLL cells (Figures 4A,C). In contrast to this effect, TQ in a low concentration slightly increased the levels of p-NF-κB p65 in LAMA-84r cells (Figure 4A). Sethi et al., 2008 have found that TQ inhibited NF-κB in CML cells in experiments with incubation times up to 6 h (Sethi et al., 2008). Metformin have shown an NF-κB inhibitory activity in various cancer cells (Nguyen et al., 2019; Sultuybek et al., 2019; Yenmis et al., 2021). However, there are controversial results regarding metformin activity against phosphorylation of Akt in leukemia cells and this activity might depend on the mitochondrial energetic status and basal levels of Akt in each cell line tested (Scotland et al., 2013). In our study, metformin decreased levels of p-Akt in LAMA-84r cell lines while in LAMA-84s cell lines, this effect could not be observed because of very low or undetectable basal levels of p-Akt. Specifically, TQ in lower concentrations induced phosphorylation of Akt while higher concentrations inhibited p-Akt. TQ stimulatory effects on p-Akt were previously observed in MDA-MB-231 breast cancer cells (Yu and Kim, 2012) while the inhibition of p-Akt by TQ was previously confirmed in various cancer models (Koka et al., 2008; Yi et al., 2008; Arafa et al., 2011; Hussain et al., 2011; Rajput et al., 2013; Khan et al., 2019). The observed TQ hormetic effects where low concentration increased levels of p-NF-κB p65 in LAMA-84r cells and p-Akt in both LAMA-84 cell lines were probably due to compensatory mechanisms activated by cells. Increased Akt phosphorylation is recognized as a compensatory mechanism in cancer cell lines (Bergholz and Zhao, 2021). In some western blot experiments, we have added doxorubicin, which induces p-Akt in several cancer cell lines such as T-lymphoblastic leukemia (Maraldi et al., 2011), breast cancer (Wallin et al., 2010), gastric cancer (Zhou et al., 2013), and osteosarcoma (Wang et al., 2015). We have introduced this specific experimental design since it was previously seen that TQ increases anti-tumor effects of doxorubicin (Effenberger-Neidnicht and Schobert, 2011). In LAMA-84s cell lines, doxorubicin did not induce the phosphorylation of Akt. Primary CLL cells were more sensitive to metformin and TQ treatment compared to cell lines, and no hormetic effects were observed. Proteins involved in proliferation, p-Akt, and p-NF-κB p65 were decreased while cleaved PARP was increased when treated with very low concentrations of metformin and TQ, especially in combination. Compensatory response is not often seen in primary cells and it is more characteristic for cancer cell lines due to various changes during adaptation to *in vitro* growth conditions (Goodspeed et al., 2016).

In conclusion, this study has shown that metformin and TQ monotherapies possess a significant anti-leukemic effect that is more pronounced when combinatorial therapies are applied. Synergistic effects on the inhibition of proliferative capacity, particularly seen in imatinib-resistant leukemic cells, represent an encouraging direction for prospective *in vivo* studies.

## Data Availability

The original contributions presented in the study are included in the article/Supplementary Material, further inquiries can be directed to the corresponding author.

## References

[B1] Al-Akra L. Bae D.-H. Leck L. Y. W. Richardson D. R. Jansson P. J. (2019). The Biochemical and Molecular Mechanisms Involved in the Role of Tumor Micro-environment Stress in Development of Drug Resistance. Biochim. Biophys. Acta (Bba) - Gen. Subjects 1863, 1390–1397. 10.1016/j.bbagen.2019.06.007 31202693

[B2] Alhosin M. Abusnina A. Achour M. Sharif T. Muller C. Peluso J. (2010). Induction of Apoptosis by Thymoquinone in Lymphoblastic Leukemia Jurkat Cells Is Mediated by a P73-dependent Pathway Which Targets the Epigenetic Integrator UHRF1. Biochem. Pharmacol. 79, 1251–1260. 10.1016/j.bcp.2009.12.015 20026309

[B3] Arafa el-S. A. Zhu Q. Shah Z. I. Wani G. Barakat B. M. Racoma I. (2011). Thymoquinone Up-Regulates PTEN Expression and Induces Apoptosis in Doxorubicin-Resistant Human Breast Cancer Cells. Mutat. Res. 706, 28–35. 10.1016/j.mrfmmm.2010.10.007 21040738 PMC3037029

[B4] Bao B. Ahmad A. Azmi A. S. Ali S. Sarkar F. H. (2013). Overview of Cancer Stem Cells (CSCs) and Mechanisms of Their Regulation: Implications for Cancer Therapy. Curr. Protoc. Pharmacol. 14. 61 Unit-14.25. 10.1002/0471141755.ph1425s61 PMC3733496 23744710

[B5] Bednar F. Simeone D. M. (2012). Metformin and Cancer Stem Cells: Old Drug, New Targets. Cancer Prev. Res. (Phila) 5, 351–354. 10.1158/1940-6207.CAPR-12-0026 22389436

[B6] Bergholz J. S. Zhao J. J. (2021). How Compensatory Mechanisms and Adaptive Rewiring Have Shaped Our Understanding of Therapeutic Resistance in Cancer. Cancer Res. 81, 6074–6077. 10.1158/0008-5472.CAN-21-3605 34911779 PMC9033251

[B7] Bhaskar A. Raturi K. Dang S. Gabrani R. (2014). Current Perspectives on the Therapeutic Aspects of Chronic Myelogenous Leukemia. Expert Opin. Ther. Pat. 24, 1117–1127. 10.1517/13543776.2014.953056 25162589

[B8] Bijnsdorp I. V. Giovannetti E. Peters G. J. (2011). Analysis of Drug Interactions. Methods Mol. Biol. 731, 421–434. 10.1007/978-1-61779-080-5_34 21516426

[B9] Calabrese E. J. Mattson M. P. (2017). How Does Hormesis Impact Biology, Toxicology, and Medicine? NPJ Aging Mech. Dis. 3, 13. 10.1038/s41514-017-0013-z 28944077 PMC5601424

[B10] Chen H. Sun B. Sun H. Xu L. Wu G. Tu Z. (2021). Bak Instead of Bax Plays a Key Role in Metformin-Induced Apoptosis S in HCT116 Cells. Cell Death Discov 7, 363. 10.1038/s41420-021-00755-y 34811352 PMC8608863

[B11] Chereda B. Melo J. V. (2016). “The Biology and Pathogenesis of Chronic Myeloid Leukemia,” in *Chronic Myeloid Leukemia* Hematologic Malignancies. Editor Hehlmann R. (Cham: Springer International Publishing), 17–39. 10.1007/978-3-319-33198-0_2

[B12] Chou T. C. Talalay P. (1984). Quantitative Analysis of Dose-Effect Relationships: the Combined Effects of Multiple Drugs or Enzyme Inhibitors. Adv. Enzyme Regul. 22, 27–55. 10.1016/0065-2571(84)90007-4 6382953

[B13] Clarke M. F. (2019). Clinical and Therapeutic Implications of Cancer Stem Cells. N. Engl. J. Med. 380, 2237–2245. 10.1056/NEJMra1804280 31167052

[B14] Cortes J. Deininger M. (2006). Chronic Myeloid Leukemia. Boca Raton: CRC Press.

[B15] Curvello R. Neto M. D. S. Ramos S. P. Bin M. E. L. Shishido S. M. de Souza A. C. S. (2013). Metformin Promotes Cancer Cells Death, Inhibits PGP Expression and Sensitize MDR Leukemic Cells to the Effects of Imatinib Mesylate. Ann. Oncol. 24, i23–i24. 10.1093/annonc/mdt045.5

[B16] Dergarabetian E. M. Ghattass K. I. El-Sitt S. B. Al-Mismar R. M. El-Baba C. O. Itani W. S. (2013). Thymoquinone Induces Apoptosis in Malignant T-Cells via Generation of ROS. Front. Biosci. Elite Ed. 5, 706–719. 10.2741/e651 23277025

[B17] Dewanjee S. Dua T. K. Bhattacharjee N. Das A. Gangopadhyay M. Khanra R. (2017). Natural Products as Alternative Choices for P-Glycoprotein (P-Gp) Inhibition. Molecules 22. 10.3390/molecules22060871 PMC6152721 28587082

[B18] Diab-Assaf M. Semaan J. El-Sabban M. Al Jaouni S. K. Azar R. Kamal M. A. (2018). Inhibition of Proliferation and Induction of Apoptosis by Thymoquinone via Modulation of TGF Family, P53, P21 and Bcl-2α in Leukemic Cells. Anticancer Agents Med. Chem. 18 (2), 210–215. 10.2174/1871520617666170912133054 28901264

[B19] Dowling R. J. Goodwin P. J. Stambolic V. (2011). Understanding the Benefit of Metformin Use in Cancer Treatment. BMC Med. 9, 33. 10.1186/1741-7015-9-33 21470407 PMC3224599

[B20] Effenberger-Neidnicht K. Schobert R. (2011). Combinatorial Effects of Thymoquinone on the Anti-cancer Activity of Doxorubicin. Cancer Chemother. Pharmacol. 67, 867–874. 10.1007/s00280-010-1386-x 20582416

[B21] El-Mahdy M. A. Zhu Q. Wang Q. E. Wani G. Wani A. A. (2005). Thymoquinone Induces Apoptosis through Activation of Caspase-8 and Mitochondrial Events in P53-Null Myeloblastic Leukemia HL-60 Cells. Int. J. Cancer 117, 409–417. 10.1002/ijc.21205 15906362

[B22] Fontaine E. (2018). Metformin-Induced Mitochondrial Complex I Inhibition: Facts, Uncertainties, and Consequences. Front. Endocrinol. (Lausanne) 9, 753. 10.3389/fendo.2018.00753 30619086 PMC6304344

[B23] Fontana S. Alessandro R. Barranca M. Giordano M. Corrado C. Zanella-Cleon I. (2007). Comparative Proteome Profiling and Functional Analysis of Chronic Myelogenous Leukemia Cell Lines. J. Proteome Res. 6, 4330–4342. 10.1021/pr0704128 17935311

[B24] García-Heredia J. M. Carnero A. (2020). Role of Mitochondria in Cancer Stem Cell Resistance. Cells 9, 1693. 10.3390/cells9071693 PMC7407626 32679735

[B25] Gatenby R. Brown J. (2018). The Evolution and Ecology of Resistance in Cancer Therapy. Cold Spring Harb. Perspect. Med. 8, a033415. 10.1101/cshperspect.a033415 28710258 PMC5830903

[B26] Goodspeed A. Heiser L. M. Gray J. W. Costello J. C. (2016). Tumor-Derived Cell Lines as Molecular Models of Cancer Pharmacogenomics. Mol. Cancer Res. 14, 3–13. 10.1158/1541-7786.MCR-15-0189 26248648 PMC4828339

[B27] Gopalakrishnapillai A. Kolb E. A. Barwe S. (2014). Metformin Suppresses Pediatric Acute Myeloid Leukemia Cell Viability and Clonogenicity. Cancer Metab. 2, P23. 10.1186/2049-3002-2-S1-P23

[B28] Green A. S. Chapuis N. Maciel T. T. Willems L. Lambert M. Arnoult C. (2010). The LKB1/AMPK Signaling Pathway Has Tumor Suppressor Activity in Acute Myeloid Leukemia through the Repression of mTOR-dependent Oncogenic mRNA Translation. Blood 116, 4262–4273. 10.1182/blood-2010-02-269837 20668229

[B29] Grimaldi C. Chiarini F. Tabellini G. Ricci F. Tazzari P. L. Battistelli M. (2012). AMP-dependent Kinase/mammalian Target of Rapamycin Complex 1 Signaling in T-Cell Acute Lymphoblastic Leukemia: Therapeutic Implications. Leukemia 26, 91–100. 10.1038/leu.2011.269 21968881

[B30] Grønningsæter I. S. Reikvam H. Aasebø E. Bartaula-Brevik S. Tvedt T. H. Bruserud Ø. (2020). Targeting Cellular Metabolism in Acute Myeloid Leukemia and the Role of Patient Heterogeneity. Cells 9, 1155. 10.3390/cells9051155 PMC7290417 32392896

[B31] Günes-Bayir A. Kocyigit A. Guler E. M. Dadak A. (2020). *In Vitro* Hormetic Effect Investigation of Thymol on Human Fibroblast and Gastric Adenocarcinoma Cells. Molecules 25, E3270. 10.3390/molecules25143270 32709059 PMC7397309

[B32] Hussain A. R. Ahmed M. Ahmed S. Manogaran P. Platanias L. C. Alvi S. N. (2011). Thymoquinone Suppresses Growth and Induces Apoptosis via Generation of Reactive Oxygen Species in Primary Effusion Lymphoma. Free Radic. Biol. Med. 50, 978–987. 10.1016/j.freeradbiomed.2010.12.034 21215312

[B33] Jabbour E. Parikh S. A. Kantarjian H. Cortes J. (2011). Chronic Myeloid Leukemia: Mechanisms of Resistance and Treatment. Hematol. Oncol. Clin. North. Am. 25, 981–v. 10.1016/j.hoc.2011.09.004 22054730 PMC4428141

[B34] Jang S. K. Hong S. E. Lee D. H. Kim J. Y. Kim J. Y. Hong J. (2021). Inhibition of AKT Enhances the Sensitivity of NSCLC Cells to Metformin. Anticancer Res. 41, 3481–3487. 10.21873/anticanres.15135 34230143

[B89] Janjetovic K. Vucicevic L. Misirkic M. Vilimanovich U. Tovilovic G. Zogovic N. (2011). Metformin Reduces Cisplatin-Mediated Apoptotic Death of Cancer Cells Through AMPK-Independent Activation of Akt. Eur. J. Pharmacol. 651 (1‐3), 41–50. 21114978 10.1016/j.ejphar.2010.11.005

[B35] Jukić M. Miloš M. (2005). Catalytic Oxidation and Antioxidant Properties of Thyme Essential Oils (Thymus Vulgarae L.). Croat. Chem. Acta 78, 105–110.

[B36] Kangwan N. Park J. M. Kim E. H. Hahm K. B. (2014). Chemoquiescence for Ideal Cancer Treatment and Prevention: where Are We Now? J. Cancer Prev. 19, 89–96. 10.15430/JCP.2014.19.2.89 25337576 PMC4204166

[B37] Kanigur Sultuybek G. Soydas T. Yenmis G. (2019). NF-κB as the Mediator of Metformin's Effect on Ageing and Ageing-Related Diseases. Clin. Exp. Pharmacol. Physiol. 46, 413–422. 10.1111/1440-1681.13073 30754072

[B38] Khalife R. El-Hayek S. Stephany el-H. Tarras O. Hodroj M. H. Rizk S. (2014). Antiproliferative and Proapoptotic Effects of Topotecan in Combination with Thymoquinone on Acute Myelogenous Leukemia. Clin. Lymphoma Myeloma Leuk. 14 (Suppl. l), S46–S55. 10.1016/j.clml.2014.04.014 25486955

[B39] Khan A. Aldebasi Y. H. Alsuhaibani S. A. Khan M. A. (2019). Thymoquinone Augments Cyclophosphamide-Mediated Inhibition of Cell Proliferation in Breast Cancer Cells. Asian Pac. J. Cancer Prev. 20, 1153–1160. 10.31557/APJCP.2019.20.4.1153 31030489 PMC6948875

[B40] Koka P. Ali M. Mondal D. Abdel-Mageed A. Agrawal K. (2008). Thymoquinone Induced Apoptosis in PC3 Cells Involves Generation of Mitochondrial Reactive Oxygen Species (ROS) and Inhibition of Akt Phosphorylation. Cancer Res. 68, 1241.

[B41] Kubuschok B. Trepel M. (2017). Learning from the Failures of Drug Discovery in B-Cell Non-hodgkin Lymphomas and Perspectives for the Future: Chronic Lymphocytic Leukemia and Diffuse Large B-Cell Lymphoma as Two Ends of a Spectrum in Drug Development. Expert Opin. Drug Discov. 12, 733–745. 10.1080/17460441.2017.1329293 28494631

[B42] Leclerc G. M. Leclerc G. J. Kuznetsov J. N. DeSalvo J. Barredo J. C. (2013). Metformin Induces Apoptosis through AMPK-dependent Inhibition of UPR Signaling in ALL Lymphoblasts. PLoS One 8, e74420. 10.1371/journal.pone.0074420 24009772 PMC3751848

[B43] Leng W. Jiang J. Chen B. Wu Q. (2021). Metformin and Malignant Tumors: Not over the Hill. Dmso 14, 3673–3689. 10.2147/DMSO.S326378 PMC8380287 34429626

[B44] Lue J. K. Amengual J. E. O'Connor O. A. (2015). Epigenetics and Lymphoma: Can We Use Epigenetics to Prime or Reset Chemoresistant Lymphoma Programs? Curr. Oncol. Rep. 17, 40. 10.1007/s11912-015-0464-y 26141799

[B45] Maraldi T. Bertacchini J. Benincasa M. Guida M. De Pol A. Liotta L. A. (2011). Reverse-phase Protein Microarrays (RPPA) as a Diagnostic and Therapeutic Guide in Multidrug Resistant Leukemia. Int. J. Oncol. 38, 427–435. 10.3892/ijo.2010.850 21132263

[B46] Martinez Marignac V. L. Smith S. Toban N. Bazile M. Aloyz R. (2013). Resistance to Dasatinib in Primary Chronic Lymphocytic Leukemia Lymphocytes Involves AMPK-Mediated Energetic Re-programming. Oncotarget 4, 2550–2566. 10.18632/oncotarget.1508 24334291 PMC3926848

[B47] Mauro C. Leow S. C. Anso E. Rocha S. Thotakura A. K. Tornatore L. (2011). NF-κB Controls Energy Homeostasis and Metabolic Adaptation by Upregulating Mitochondrial Respiration. Nat. Cell Biol. 13, 1272–1279. 10.1038/ncb2324 21968997 PMC3462316

[B48] Minassian L. M. Cotechini T. Huitema E. Graham C. H. (2019). Hypoxia-Induced Resistance to Chemotherapy in Cancer. Adv. Exp. Med. Biol. 1136, 123–139. 10.1007/978-3-030-12734-3_9 31201721

[B49] Mu G. G. Zhang L. L. Li H. Y. Liao Y. Yu H. G. (2015). Thymoquinone Pretreatment Overcomes the Insensitivity and Potentiates the Antitumor Effect of Gemcitabine through Abrogation of Notch1, PI3K/Akt/mTOR Regulated Signaling Pathways in Pancreatic Cancer. Dig. Dis. Sci. 60, 1067–1080. 10.1007/s10620-014-3394-x 25344906

[B50] Mu H. Zhu X. Jia H. Zhou L. Liu H. (2021). Combination Therapies in Chronic Myeloid Leukemia for Potential Treatment-free Remission: Focus on Leukemia Stem Cells and Immune Modulation. Front. Oncol. 11, 1657. 10.3389/fonc.2021.643382 PMC8155539 34055612

[B51] Neuzil J. Stantic M. Zobalova R. Chladova J. Wang X. Prochazka L. (2007). Tumour-initiating Cells vs. Cancer 'stem' Cells and CD133: What's in the Name? Biochem. Biophys. Res. Commun. 355, 855–859. 10.1016/j.bbrc.2007.01.159 17307142

[B52] Nguyen T. T. Ung T. T. Li S. Lian S. Xia Y. Park S. Y. (2019). Metformin Inhibits Lithocholic Acid-Induced Interleukin 8 Upregulation in Colorectal Cancer Cells by Suppressing ROS Production and NF-kB Activity. Sci. Rep. 9, 2003. 10.1038/s41598-019-38778-2 30765814 PMC6376015

[B53] Pang J. Shen N. Yan F. Zhao N. Dou L. Wu L. C. (2017). Thymoquinone Exerts Potent Growth-Suppressive Activity on Leukemia through DNA Hypermethylation Reversal in Leukemia Cells. Oncotarget 8, 34453–34467. 10.18632/oncotarget.16431 28415607 PMC5470982

[B54] Park S. Willingham M. C. Qi J. Cheng S. Y. (2018). Metformin and JQ1 Synergistically Inhibit Obesity-Activated Thyroid Cancer. Endocr. Relat. Cancer 25, 865–877. 10.1530/ERC-18-0071 29914872 PMC6059993

[B55] Radujkovic A. Luft T. Dreger P. Ho A. D. Jens Zeller W. Fruehauf S. (2014). *In Vitro* testing of Drug Combinations Employing Nilotinib and Alkylating Agents with Regard to Pretransplant Conditioning Treatment of Advanced-phase Chronic Myeloid Leukemia. Cancer Chemother. Pharmacol. 74, 427–432. 10.1007/s00280-014-2533-6 25038611

[B56] Radujkovic A. Schad M. Topaly J. Veldwijk M. R. Laufs S. Schultheis B. S. (2005). Synergistic Activity of Imatinib and 17-AAG in Imatinib-Resistant CML Cells Overexpressing BCR-ABL--Inhibition of P-Glycoprotein Function by 17-AAG. Leukemia 19, 1198–1206. 10.1038/sj.leu.2403764 15902298

[B57] Rajput S. Kumar B. N. Sarkar S. Das S. Azab B. Santhekadur P. K. (2013). Targeted Apoptotic Effects of Thymoquinone and Tamoxifen on XIAP Mediated Akt Regulation in Breast Cancer. PLoS One 8, e61342. 10.1371/journal.pone.0061342 23613836 PMC3629226

[B58] Reddy M. M. Nonami A. Weisberg E. L. Xing K. Salgia S. K. Asara J. M. (2013). Small Molecule Activators of AMPK Block the Glycogen Production Required for Transformation of Myeloid Leukemia Cells. Blood 122, 1479. 10.1182/blood.V122.21.1479.1479

[B59] Rosilio C. Ben-Sahra I. Bost F. Peyron J. F. (2014). Metformin: a Metabolic Disruptor and Anti-diabetic Drug to Target Human Leukemia. Cancer Lett. 346, 188–196. 10.1016/j.canlet.2014.01.006 24462823

[B60] Rosilio C. Lounnas N. Nebout M. Imbert V. Hagenbeek T. Spits H. (2013). The Metabolic Perturbators Metformin, Phenformin and AICAR Interfere with the Growth and Survival of Murine PTEN-Deficient T Cell Lymphomas and Human T-ALL/T-LL Cancer Cells. Cancer Lett. 336, 114–126. 10.1016/j.canlet.2013.04.015 23612073

[B61] Salim L. Z. Mohan S. Othman R. Abdelwahab S. I. Kamalidehghan B. Sheikh B. Y. (2013). Thymoquinone Induces Mitochondria-Mediated Apoptosis in Acute Lymphoblastic Leukaemia *In Vitro* . Molecules 18, 11219–11240. 10.3390/molecules180911219 24036512 PMC6269888

[B62] Salim L. Z. Othman R. Abdulla M. A. Al-Jashamy K. Ali H. M. Hassandarvish P. (2014). Thymoquinone Inhibits Murine Leukemia WEHI-3 Cells *In Vivo* and *In Vitro* . PLOS ONE 9, e115340. 10.1371/journal.pone.0115340 25531768 PMC4274020

[B63] Sang F. Ding Y. Wang J. Sun B. Sun J. Geng Y. (2016). Structure-Activity Relationship Study of Rakicidins: Overcoming Chronic Myeloid Leukemia Resistance to Imatinib with 4-Methylester-Rakicidin A. J. Med. Chem. 59, 1184–1196. 10.1021/acs.jmedchem.5b01841 26814890

[B64] Saraei P. Asadi I. Kakar M. A. Moradi-Kor N. (2019). The Beneficial Effects of Metformin on Cancer Prevention and Therapy: a Comprehensive Review of Recent Advances. Cancer Manag. Res. 11, 3295–3313. 10.2147/CMAR.S200059 31114366 PMC6497052

[B65] Scappini B. Gatto S. Onida F. Ricci C. Divoky V. Wierda W. G. (2004). Changes Associated with the Development of Resistance to Imatinib (STI571) in Two Leukemia Cell Lines Expressing P210 Bcr/Abl Protein. Cancer 100, 1459–1471. 10.1002/cncr.20131 15042680

[B66] Scotland S. Saland E. Skuli N. de Toni F. Boutzen H. Micklow E. (2013). Mitochondrial Energetic and AKT Status Mediate Metabolic Effects and Apoptosis of Metformin in Human Leukemic Cells. Leukemia 27, 2129–2138. 10.1038/leu.2013.107 23568147 PMC10869165

[B67] Sethi G. Ahn K. S. Aggarwal B. B. (2008). Targeting Nuclear Factor-Kappa B Activation Pathway by Thymoquinone: Role in Suppression of Antiapoptotic Gene Products and Enhancement of Apoptosis. Mol. Cancer Res. 6, 1059–1070. 10.1158/1541-7786.MCR-07-2088 18567808

[B68] Shi R. Lin J. Gong Y. Yan T. Shi F. Yang X. (2015). The Antileukemia Effect of Metformin in the Philadelphia Chromosome-Positive Leukemia Cell Line and Patient Primary Leukemia Cell. Anticancer. Drugs 26, 913–922. 10.1097/CAD.0000000000000266 26186064

[B69] Shi W. Y. Xiao D. Wang L. Dong L. H. Yan Z. X. Shen Z. X. (2012). Therapeutic Metformin/AMPK Activation Blocked Lymphoma Cell Growth via Inhibition of mTOR Pathway and Induction of Autophagy. Cell Death Dis 3, e275. 10.1038/cddis.2012.13 22378068 PMC3317343

[B70] Siegfried Z. Karni R. (2018). The Role of Alternative Splicing in Cancer Drug Resistance. Curr. Opin. Genet. Dev. 48, 16–21. 10.1016/j.gde.2017.10.001 29080552

[B71] Siveen K. S. Mustafa N. Li F. Kannaiyan R. Ahn K. S. Kumar A. P. (2014). Thymoquinone Overcomes Chemoresistance and Enhances the Anticancer Effects of Bortezomib through Abrogation of NF-Κb Regulated Gene Products in Multiple Myeloma Xenograft Mouse Model. Oncotarget 5, 634–648. 10.18632/oncotarget.1596 24504138 PMC3996662

[B72] Skoda J. Borankova K. Jansson P. J. Huang M. L. Veselska R. Richardson D. R. (2019). Pharmacological Targeting of Mitochondria in Cancer Stem Cells: An Ancient Organelle at the Crossroad of Novel Anti-cancer Therapies. Pharmacol. Res. 139, 298–313. 10.1016/j.phrs.2018.11.020 30453033

[B73] Soltani A. Pourgheysari B. Shirzad H. Sourani Z. (2017). Antiproliferative and Apoptosis-Inducing Activities of Thymoquinone in Lymphoblastic Leukemia Cell Line. Indian J. Hematol. Blood Transfus. 33, 516–524. 10.1007/s12288-016-0758-8 29075062 PMC5640521

[B74] Taniguchi C. M. Emanuelli B. Kahn C. R. (2006). Critical Nodes in Signalling Pathways: Insights into Insulin Action. Nat. Rev. Mol. Cell Biol. 7, 85–96. 10.1038/nrm1837 16493415

[B75] Vakana E. Altman J. K. Glaser H. Donato N. J. Platanias L. C. (2011). Antileukemic Effects of AMPK Activators on BCR-ABL-Expressing Cells. Blood 118, 6399–6402. 10.1182/blood-2011-01-332783 22021366 PMC3236122

[B76] Valiulienė G. Vitkevičienė A. Skliutė G. Borutinskaitė V. Navakauskienė R. (2021). Pharmaceutical Drug Metformin and MCL1 Inhibitor S63845 Exhibit Anticancer Activity in Myeloid Leukemia Cells via Redox Remodeling. Molecules 26. 10.3390/molecules26082303 PMC8071510 33921161

[B77] Voltan R. Rimondi E. Melloni E. Gilli P. Bertolasi V. Casciano F. (2016). Metformin Combined with Sodium Dichloroacetate Promotes B Leukemic Cell Death by Suppressing Anti-apoptotic Protein Mcl-1. Oncotarget 7, 18965–18977. 10.18632/oncotarget.7879 26959881 PMC4951344

[B78] Wallin J. J. Guan J. Prior W. W. Edgar K. A. Kassees R. Sampath D. (2010). Nuclear Phospho-Akt Increase Predicts Synergy of PI3K Inhibition and Doxorubicin in Breast and Ovarian Cancer. Sci. Transl. Med. 2, 48ra66. 10.1126/scitranslmed.3000630 20826841

[B79] Wang Z. Yang L. Xia Y. Guo C. Kong L. (2015). Icariin Enhances Cytotoxicity of Doxorubicin in Human Multidrug-Resistant Osteosarcoma Cells by Inhibition of ABCB1 and Down-Regulation of the PI3K/Akt Pathway. Biol. Pharm. Bull. 38, 277–284. 10.1248/bpb.b14-00663 25747987

[B80] Ye H. Liu Y. Wu K. Luo H. Cui L. (2020). AMPK Activation Overcomes Anti-EGFR Antibody Resistance Induced by KRAS Mutation in Colorectal Cancer. Cell Commun Signal 18, 115. 10.1186/s12964-020-00584-z 32703218 PMC7376720

[B81] Yenmis G. Yaprak Sarac E. Besli N. Soydas T. Tastan C. Dilek Kancagi D. (2021). Anti-cancer Effect of Metformin on the Metastasis and Invasion of Primary Breast Cancer Cells through Mediating NF-kB Activity. Acta Histochem. 123, 151709. 10.1016/j.acthis.2021.151709 33711726

[B82] Yi T. Cho S. G. Yi Z. Pang X. Rodriguez M. Wang Y. (2008). Thymoquinone Inhibits Tumor Angiogenesis and Tumor Growth through Suppressing AKT and Extracellular Signal-Regulated Kinase Signaling Pathways. Mol. Cancer Ther. 7, 1789–1796. 10.1158/1535-7163.MCT-08-0124 18644991 PMC2587125

[B83] Yu S. M. Kim S. J. (2012). Thymoquinone (TQ) Regulates Cyclooxygenase-2 Expression and Prostaglandin E2 Production through PI3kinase (PI3K)/p38 Kinase Pathway in Human Breast Cancer Cell Line, MDA-MB-231. Anim. Cell Syst. 16, 274–279. 10.1080/19768354.2011.647834

[B84] Yuan F. Cheng C. Xiao F. Liu H. Cao S. Zhou G. (2020). Inhibition of mTORC1/P70S6K Pathway by Metformin Synergistically Sensitizes Acute Myeloid Leukemia to Ara-C. Life Sci. 243, 117276. 10.1016/j.lfs.2020.117276 31926250

[B85] Zhang L. Bai Y. Yang Y. (2016). Thymoquinone Chemosensitizes colon Cancer Cells through Inhibition of NF-Κb. Oncol. Lett. 12, 2840–2845. 10.3892/ol.2016.4971 27698868 PMC5038441

[B86] Zhang X. Fu X. Dong M. Yang Z. Wu S. Ma M. (2018). Conserved Cell Populations in Doxorubicin-Resistant Human Nasal Natural Killer/T Cell Lymphoma Cell Line: Super Multidrug Resistant Cells? Cancer Cell Int 18, 150. 10.1186/s12935-018-0644-6 30302057 PMC6167813

[B87] Zhou F. J. Zeng C. X. Kuang W. Cheng C. Liu H. C. Yan X. Y. (2021). Metformin Exerts a Synergistic Effect with Venetoclax by Downregulating Mcl-1 Protein in Acute Myeloid Leukemia. J. Cancer 12, 6727–6739. 10.7150/jca.60208 34659562 PMC8518002

[B88] Zhou W. Fu X. Q. Zhang L. L. Zhang J. Huang X. Lu X. H. (2013). The AKT1/NF-kappaB/Notch1/PTEN axis Has an Important Role in Chemoresistance of Gastric Cancer Cells. Cell Death Dis 4, e847. 10.1038/cddis.2013.375 24113181 PMC3824684

